# Computer-aided detection system for lung cancer in computed tomography scans: Review and future prospects

**DOI:** 10.1186/1475-925X-13-41

**Published:** 2014-04-08

**Authors:** Macedo Firmino, Antônio H Morais, Roberto M Mendoça, Marcel R Dantas, Helio R Hekis, Ricardo Valentim

**Affiliations:** 1 Department of Information and Computer Science, Federal Institute of Rio Grande do Norte (IFRN), Natal, Brazil; 2 Department of Radiology and Medical Imaging - University Hospital Onofre Lopes (HUOL), Natal, Brazil; 3 Laboratory of Technological Innovation in Healthcare, Federal University of Rio Grande do Norte (UFRN), Natal, Brazil

**Keywords:** Computer-aided detection system, Lung cancer diagnosis, Medical image analysis, Detection of pulmonary nodules, CADe systems survey

## Abstract

**Introduction:**

The goal of this paper is to present a critical review of major Computer-Aided Detection systems (CADe) for lung cancer in order to identify challenges for future research. CADe systems must meet the following requirements: improve the performance of radiologists providing high sensitivity in the diagnosis, a low number of false positives (FP), have high processing speed, present high level of automation, low cost (of implementation, training, support and maintenance), the ability to detect different types and shapes of nodules, and software security assurance.

**Methods:**

The relevant literature related to “CADe for lung cancer” was obtained from PubMed, IEEEXplore and Science Direct database. Articles published from 2009 to 2013, and some articles previously published, were used. A systemic analysis was made on these articles and the results were summarized.

**Discussion:**

Based on literature search, it was observed that many if not all systems described in this survey have the potential to be important in clinical practice. However, no significant improvement was observed in sensitivity, number of false positives, level of automation and ability to detect different types and shapes of nodules in the studied period. Challenges were presented for future research.

**Conclusions:**

Further research is needed to improve existing systems and propose new solutions. For this, we believe that collaborative efforts through the creation of open source software communities are necessary to develop a CADe system with all the requirements mentioned and with a short development cycle. In addition, future CADe systems should improve the level of automation, through integration with picture archiving and communication systems (PACS) and the electronic record of the patient, decrease the number of false positives, measure the evolution of tumors, evaluate the evolution of the oncological treatment, and its possible prognosis.

## Introduction

Cancer is a group of diseases characterized by maturation, growth and/or disorganized proliferation of abnormal cell groups [1]. According to the World Health Organization [2], cancer is a leading cause of death worldwide. In the U.S alone, 1,660,290 new cases and 580,350 deaths from the disease are estimated for the year of 2013 [3]. Lung cancer is one of the most common cancers [4], with estimated 228,190 new cases and 159,480 deaths in the U.S. alone in 2013 [3]. One way to try to minimize this high mortality rate is through early detection and treatment. Recently, advances in computed tomography (CT) has allowed early diagnosis of the disease [5]. According to Awai et al. [6] the detection rate of lung cancer using CT is 2.6 to 10 times higher than by using analog radiography. However, the use of CT is directly impacting the workload of radiologists who need to analyze an increasing number of screening tests in a short time. This workload can result in errors in detection (failure to detect) or misinterpretation (inability to properly diagnose a tumor). Therefore, computational systems are needed to assist radiologists in the interpretation of images, nodule detection and determination of their characteristics are needed.

There are two main computational systems developed to assist radiologists, they are: CADe (computer-aided detection system) and CADx (computer-aided diagnosis system). CADe systems detect lesions through medical images while CADx systems aim to measure the lesion characterization, for example, determining the malignancy and staging of the cancer (CADx systems are outside the scope of this work). CADe systems have the following goals [7]: 

•Improve accuracy in diagnosis;

•Assist in early detection of cancer;

•Reduce the time of the radiologist in exam evaluation.

CADe systems are an important tool for medical radiology, however, many systems do not yet have all the necessary requirements to be considered useful by most radiologists. Among the requirements that are cited by radiologists stand out [8,9]: 

•Improve the performance of radiologists providing high sensitivity in diagnosis. The sensitivity of these systems is given by the formula:

(1) $$\begin{array}{lcrr} \text{sensitivity} & = & \frac{\text{TP}}{\left(\text{TP}+\text{FN}\right)} & \end{array}$$

•where: TP (true positive) represents the results that the system presented positively to a sample that actually had the disease, and FN (false negative) the negative results when the sample had the disease.

•A low number of false positives (FP). FP happens when the system determines the existence of the disease when the sample showed no disease. False positives result in increased reading time by radiologists and can result in errors in detection;

•Have high processing speed. This refers to the time taken for the system to respond to requests of detection;

•Present high level of automation avoiding the occurrence of manual operations. The system should automatically receive DICOM files of all examinations, undertake the processing and store the results in a standardized report.

•Present a low cost of implementation, training, support and system maintenance;

•Detect different types and shapes of nodules, e.g., solitary nodules, small nodules (< 3 mm), ground-glass opacity nodules, nodules attached to the lung borders and cavity nodules;

•Software security assurance for avoiding potential harms that could result from the loss, inaccuracy, alteration, unavailability, or misuse of the data. Security techniques are outside the scope of this work.

In 2012, seeking to prove the importance of CADe systems for radiology, Jeon et al. [10] requested seven radiologists to analyze 134 CT scans and to determine the presence of nodules. Then, the same radiologists reviewed their decisions after analyzing the results of the CADe system. As a result, the average detection rate of nodules was increased from 77% at initial evaluation to 84% with the aid of the CADe system. A parallel study was performed by Bogoni et al. [11] that evaluated the impact on efficiency of radiologists through a CADe tool (syngo LungCAD) integrated to a commercial PACS (Picture Archiving and Communication Systems), called Siemens syngo CADe Manager. Five radiologists analyzed 48 CT scans. Further, they observed the results of the CADe system. As a result, it was observed that these radiologists improved their performance in the detection of nodules with the use of the tool, as can be seen in Table 1.

**Table 1:** Comparing the Performance of five radiologists in the detection of pulmonary nodules with, and without, a CADe tool (syngo LungCAD) integrated to a commercial PACS system

**Size of nodules**	**Peformance**	
	**without CADe**	**with CADe**
≥ 3mm and ≤ 4mm	44%	57%
≥ 4mm and ≤ 5mm	48%	61%
≥ 5mm	44%	60%

Currently, even though CADe systems are proven to improve the efficiency of radiologists in the detection of nodules, they are not widely used in clinical practice [9]. As a result, CADe systems have become one of the most important areas of research in medical image processing. The purpose of this paper is to present a review of CADe systems for the detection of lung cancer in CT scans to identify challenges for future research.

There are other papers that perform a bibliographic review of systems for the detection of nodules, for example, Gomathi and Thangaraj [7], Lee et al. [12], Suzuki [13] and El Baz et al. [8]. However, Gomathi and Thangaraj [7] and Lee et al. [12] showed progress until the year of 2009 and 2010, respectively, and did not address challenges. Suzuki [13] and El Baz et al. [8] showed progress until June 2012. The current paper, however, presents an analysis of the main CADe systems for lung cancer released until August 2013 with the goal to discuss challenges for future research.

### Generic architecture of CADe systems

CADe systems for detecting pulmonary nodules are usually composed of five subsystems: acquisition, preprocessing, segmentation, nodule detection and elimination of false positives. For users of CADe systems, it is important to have a basic understanding of these subsystems in order to understand its operation. Further along, functions, characteristics and main techniques for each subsystem will be presented.

#### Acquisition

The acquisition subsystem is responsible for obtaining medical images. Often, the CADe systems are developed, trained and validated with private databases obtained from partner hospitals. However, the use of private databases hampers the comparison between different CADe systems. Bogoni et al. [11] showed that the use of a CADe system with a PACS in a hospital environment becomes more efficient for automated localization of series of CT data. A PACS consists of image acquisition devices, a data management system, image storage devices, transmission network, display stations, and devices to produce hard-copy images if required [14,15].

Public databases can be used to develop, train and validate CADe systems. It is used also for training medical students, as an archive of rare cases, and it enables the comparison of different CADe systems [16]. Public databases must have large data such as follow-up images to evaluate change over time, pathology reports, or radiologist-drawn lesion outlines [17]. Among the public databases, LIDC (Lung Image Database Consortium) stands out [17] which aims to create and maintain a database of images of pulmonary examinations. Along with the images, radiological annotations performed by professionals with extensive experience are provided. These annotations identify the location and radiological characteristics of the lesions and certain lung abnormalities. ANODE09 [18] is another public database of lung nodules that aims to provide a quantitative comparison (regarding sensitivity and number of FP) between CADe systems for detection of pulmonary nodules.

#### Preprocessing

**Figure 1:**
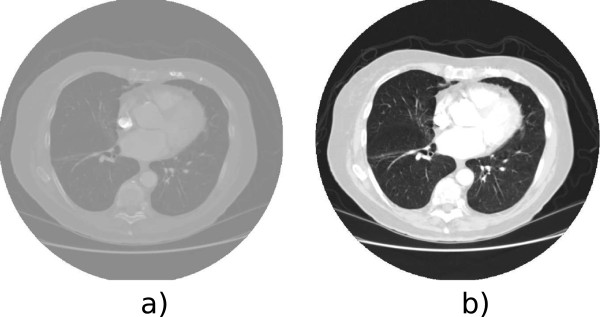
**Preprocessing of a chest CT scan.** a) original image b) image with changes on opacity, color and gradient. Removing defects caused by lack of contrast in the image acquisition process by filters of opacity, color and gradient to improve the image quality.

Preprocessing is the treatment performed on the image that aims to improve the quality of it to increase the precision and accuracy of processing algorithms that take place after this stage [7]. This stage removes defects caused by the image acquisition process, for example, noise and lack of contrast, as can be seen in Figure 1.

In this context, the main techniques for preprocessing are: Median Filtering [19], Enhancement Filter [20,21], Contrast Limited Adaptive Histogram Equalization [19], Auto-enhancement [22], Wiener filter [22], Fast Fourier Transform [23], Wavelet Transform [23], Antigeometric Diffusion [23], Erosion Filter [24], Smoothing filters [25] and Noise Correction [25].

#### Segmentation of pulmonary images

**Figure 2:**
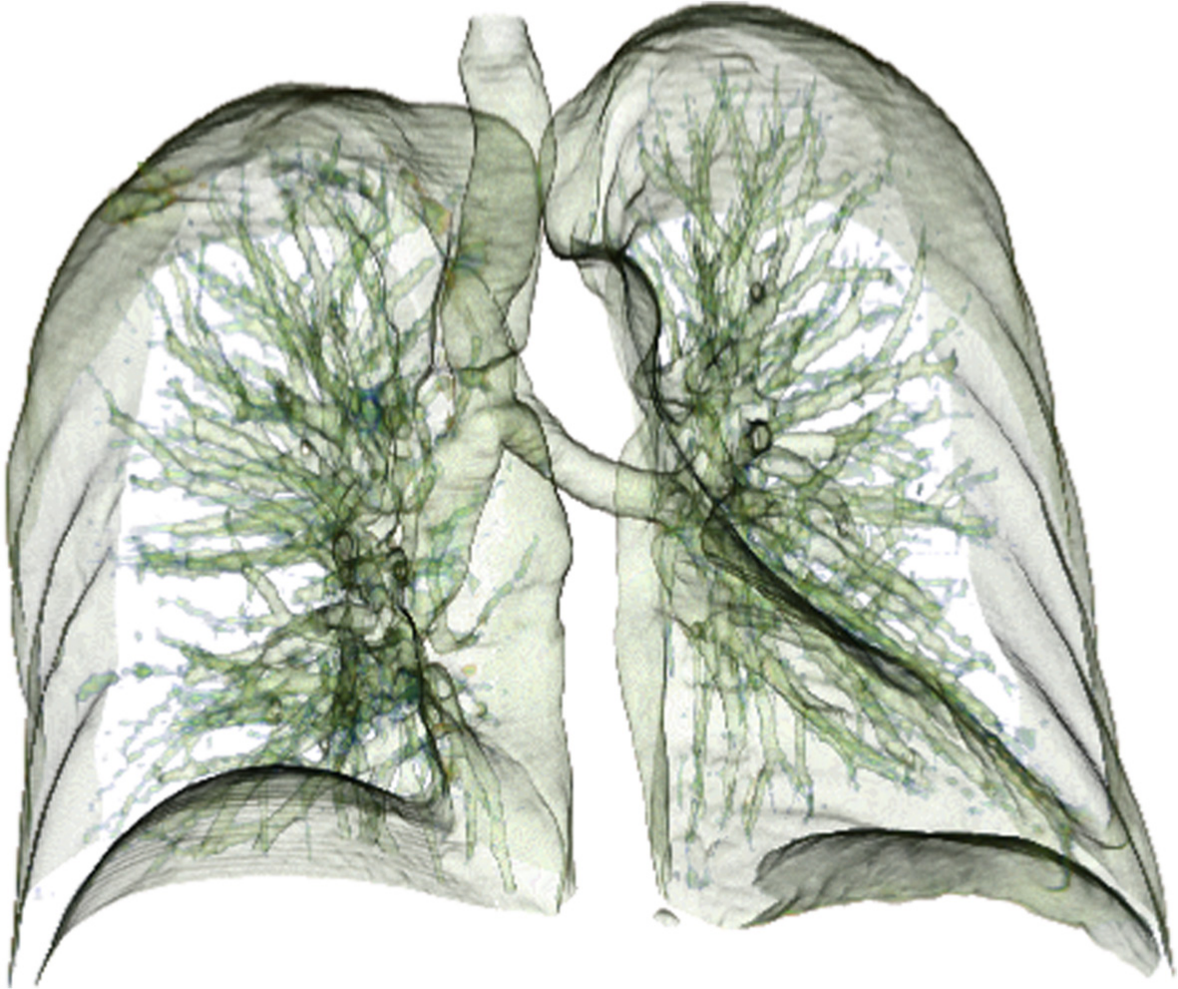
**Image of lungs segmented through the 3D Slicer tool.** Using EM Segmentation algorithm to separate the lung region from other organs and tissues on the computed tomography image with 3D slicer tools.

This subsystem has the function to separate the study region from other organs and tissues in radiographic images in order to reduce the computational cost of the next stages, as can be seen in Figure 2. The two main approaches for segmentation of lung images are: segmentation based on thresholding and segmentation by deformable models. In the segmentation approach based on thresholding, a threshold of intensity to perform the separation is utilized. This approach is possible since in the CT scans, lung tissues are present in darker shades (low values of Hounsfield Units - HU) when compared to other organs, such as heart, liver, and bone tissue [8]. Some authors calculate the threshold iteratively [26] while others use this approach in conjunction with the following methods: Otsu’s [27], morphological operations [26,28,29], rolling ball algorithm [30], edge detection algorithm [31], Connect-Component Labeling with morphological closing [32] and Gaussian antialiasing [23]. The main problem of this segmentation is that its accuracy is affected by the type of equipment that makes the acquisition and the location of nodules.

Deformable models are curves or surfaces, for segmentation in the image domain, which deform themselves according to the influence of internal (which are defined within the curve or surface itself) and external forces (which are computed from the image data) [33]. The main types of deformable models used for segmentation of lung images, are: active contours (snakes and geodesic) [34,35] and level set based deformable models [36]. The deformable model started from an initial segmentation obtained by a threshold estimated from CT data. The main disadvantages of this segmentation are its initialization process and the inability of external forces (e.g., based on the edges and levels of gray) to capture the lack of homogeneity in regions of the lung [8]. For further information read Devaki and Bhaskaran [37] that presented a literature review on computer analysis of lungs in CT scans addressing segmentation of various lungs anatomical structures.

#### Nodule detection

**Figure 3:**
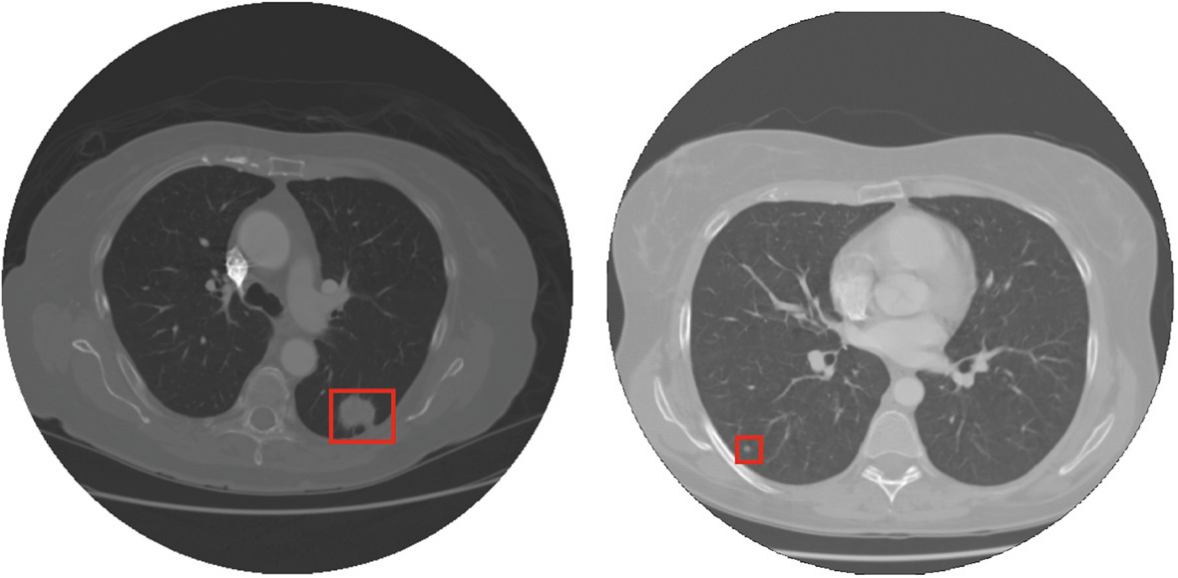
**Transverse thoracic CT images of a patient with pulmonary nodules highlighted by square: juxtapleural nodule (left) and internal nodule (right).** Computed tomography images of patients with pulmonary nodules obtained in the LIDC/IDRI Database.

The stage for nodule detection aims at determining the presence of pulmonary nodules in the image, and if this presence is detected, to inform the location of the nodules. Currently, the main difficulty for CADe systems is to distinguish true nodules from other pulmonary parenchymatous injuries or different organs and tissues. The Figure 3 shows computed tomography images of a patient with true pulmonary nodule (juxtapleural and internal) highlighted.

The main relevance of pulmonary nodules is that they often represent the initial radiographic findings of lung cancer. This disease can be divided into small cell and non-small cell carcinoma. The typical radiographic appearance of small cell carcinoma is of mediastinal lymph nodes and/or enlarged hilar, sometimes associated with pulmonary nodules. The non-small cell carcinoma can be subdivided into: adenocarcinoma, squamous cells and large cells. Adenocarcinoma is the most common type and usually appears as a solitary pulmonary nodule in the lung periphery. The squamous cell carcinoma can be shown on the radiograph as a solitary mass with cavitation or without cavitation. The large cell carcinoma is the least common type, and its appearance is of an extensive injury within the lung [38]. Nodules can be solid, semisolid and ground glass (not solid).

Accurate nodule segmentation is crucial for various diagnostic and treatment procedures for lung cancer, such as monitoring tumor response to therapy and diagnosing tumor growth and malignancy. The main sources of errors in the detection are small nodules, ground-glass opacity nodules, nodules attached to vessels (juxtavascular), and nodules attached to parenchymal wall and diaphragm (juxtapleural) [8]. Small nodules are difficult to segment due to spatial discretization used for the CT imaging where a voxel may represent more than one tissue type, resulting in averaging of their intensity values. Accurate segmentation of juxtavascular and justapleural nodules is a challenge because CT values for nodules and these non-target structures are often very similar. Ground glass nodules are difficult to detect because they are of low attenuation and have poorly defined borders. This difficulty was reported by Cascio et al. [39], who have developed a CADe system with 3D dot-enhancement filter (for nodule detection) and a neural classifier (for false-positive finding reduction) for the detection of internal and justapleural nodules. The system had a sensitivity of 100%, with 2.7 FP/scan, for internal nodes and 84.6%, with 9 FP/scan, for juxtapleural nodules.

Region growing is one of the best methods to segment tumor regions because the borders found are perfectly thin and connected [40]. Region growing method constructs regions by starting from some user provided voxels, called seeds. The region grows from this seed by comparing the values of neighboring voxels based on some user criterion, for example, pixel intensity. The disadvantage of this approach is that nodule detection is semi-automated [40]. Other techniques used for the segmentation of pulmonary nodules reported in the literature are: cylindrical and spherical filters [41-43], based on models [44-46], morphological operators [6,47], thresholding [48], multiple gray-level thresholding [49], genetic algorithm template matching of Gaussian spheres [45], clustering [50,51], connected component analysis [52], based on rules [53,54] and specific for each patient [55].

#### Elimination of false positive

This stage aims to remove the identification of false nodules through the features of the nodules found. Initially, the possible nodules detected are segmented and their features are extracted. The main extracted features are [56]: 

•Intensity values of pixels: They are extracted from the image histogram;

•Morphology: It contains information about the size and shape of the nodule. The size is determined based on the radius, area and perimeter. On the other hand the shape is determined by the compactness, roundness, smoothness, symmetry and concavity;

•Texture: It provides information on the variation in the intensity of the surface by analyzing characteristics, such as smoothness, roughness and regularity;

•Fractal: It provides information about the regularity and complexity of nodules by means of their level of self similarity.

Once the possible nodules are identified and their characteristics obtained, the CADe system tries to eliminate false positives (FP). In order to eliminate FP, classifiers are used. In general, a classification system has two phases: the classifier training to learn the parameters of the system, and the testing phase, to evaluate the success of the classifier. This approach carries the risk of data memorization consequently obtaining optimistic error rates. To circumvent these problems, it is necessary a large database of CT images.

Cross-validation is a statistical technique used to determine, during training, the generalization capability of classifiers. The training data should be divided into two distinct sets, one for training (used to train) and one for validation (used to validate). On training of classifiers for lung nodules, where have few pathological examples, a method of cross-validation called leave-one-out should be used. In this case, N - 1 examples are used to train the classifier, and the classifier is validated by testing it on the example left out. The experiment is repeated for a total of N times, each time leaving out a different example for validation [57].

The main classifiers are: linear discriminant analysis [48,58], based on rules [59,60], clustering [47], Markov random field [61], artificial neural networks [19,62], support vector machines (SVM) [20,63], massive-training neural network (MTANNs) [64], and double-threshold cut [35,65].

## Methods

The relevant literature related to “CADe for lung cancer” was obtained from PubMed, IEEEXplore and Science Direct database. A total of 420 articles were found based in keywords, single or combined, as well as their synonyms: CAD system, lung cancer, Computer-Aided Detection System, CADe, detection of pulmonary nodules, detection system, cancer detection, lung cancer in computed tomography scans, and medical image analysis. The search results were filtered and proceedings, editorials, and letters were excluded. Articles published from 2009 to 2013, and some articles previously published, were used. However, articles that omitted the number of FP, number of nodules used in validation and sensitivity were excluded. Finally, 70 articles were used in our study. A systemic analysis was made on these articles and the results were summarized.

## Review of CADe systems for detection of lung cancer

The first reports of the use of digital computers to detect lung nodules in chest radiographs occurred in 1963 with Lodwick et al. [66]. However, only in the late 80s the first CADe systems and patents for detecting lung nodules appeared [67,68]. Although interesting results have been obtained, these first attempts were not successful due to lack of computational resources and advanced image processing techniques. However, research had already showed that the use of CADe systems improved the accuracy of radiologists in the diagnosis, even with a large number of false positives [69].

Significant improvements in speed, sensitivity and reduction of false positives were obtained only in the late 90s by Xu et al. [70]. They used thresholding and artificial neural networks to select nodules and eliminate false positives, respectively. A sensitivity of 70% with 1.7 FP per image was obtained in approximately 20s. One of the problems encountered in this period was the low level of automation of systems because for scanning X-ray films, scanners were used, usually a manual process. In the same period, with the intention of improving the automation process emerged the first CADe systems for detection of pulmonary nodules that used CT images. Armato et al. [48] developed a CADe system that used thresholding techniques (to segment images of the lungs and identify possible nodules) and Linear Discriminant Analysis (to reduce the number of false positives). This system had a sensitivity of 70% with 9.6 FP per case. This system was validated with 187 solitary and juxtapleural nodules with sizes between 3.1 mm and 27.8 mm.

In 2001, Lee et al. [45] have developed a technique using genetic algorithm and template matching for detecting pulmonary nodules. False positives were eliminated through rules based on the characteristics of the nodules found. The system had a sensitivity of 72% with 25.3 false positives per case. In the validation of the system 98 nodules that possessed dimensions smaller than 10mm were used.

In 2002, Suzuki et al. [71] developed a pattern recognition technique based on an artificial neural network called MTANN to reduce the number of false positives in the detection of pulmonary nodules. This technique was able to process the CT image directly without the necessity of segmentation. A sensitivity of 80.3% with 4.8 FP per case was obtained being tested with 121 nodules (juxtavascular, hilum, ground-glass opacity and juxtapleural) with sizes between 4mm and 25mm.

In 2004, the Lung Image Database Consortium (LIDC) was created [72] to minimize one of the biggest barriers in the research of CADe systems for the detection of pulmonary nodules, that is, the lack of a database with a significant amount of exams. The LIDC was composed of American Universities with the goal of creating and maintaining a public database of chest CT images of normal patients and patients with lung cancer at various stages. This database is a useful tool for the development, training and evaluation of CADe systems for the detection of pulmonary nodules.

In 2007, Murphy et al. [40] presented a CADe system, named ISI-CAD, where images of the lungs were segmented through the region growing technique and morphological smoothing. Geometric filters and the k-nearest neighbor classifier were used to determine the candidate nodules and to eliminate false positives. The system had a sensitivity of 84% with 8.2 FP per case being tested with 268 pleural and non-pleural nodules with sizes between 2mm and 14mm.

In 2009, to improve the sensitivity of CADe systems, Ye et al. [23] proposed a new method to optimize the detection of nodules with ground-glass opacity (non-solid nodule). This method utilized fuzzy thresholding, feature maps, adaptive thresholding, rule-based classifier with support vector machine (SVM) to segment images of the lungs, selection of candidate nodules, nodule segmentation and elimination of false positives, respectively. The system had a sensitivity of 90.2% and 8.2 FP per case being validated with 220 nodules (juxtavascular, isolated, ground-glass opacity and juxtapleural) of sizes between 2mm and 20mm.

In 2010 there were several contributions. Messay, Hardie and Rogers [21] presented a CADe system using thresholding, morphological processing and Fisher Linear Discriminant to segment, detect candidate nodules and eliminate of false positives, respectively. The system obtained a sensitivity of 82.66% with 3 FP per case being validated with 143 nodules (juxtavascular, solitary, ground-glass opacity and juxtapleural), with sizes from 3mm to 30mm. Liu et al. [20] proposed an approach in which images were divided in three planes (axial, sagittal and coronal) to improve sensitivity. Thresholding with the rolling ball algorithm and dot-enhancement filter was used to segment images and identify candidate nodules, respectively. The characteristics of the nodules were extracted and used in three support vector machines to reduce false positives. They obtained a sensitivity of 97% and a rate of 4.3 FP per case. A negative point in relation to this work concerns the validation of the system in which it is merely tested with only 32 nodules, being 31 solitary nodules. Thus, it is not guaranteed that this system presents the same performance in other circumstances, because the system was not tested with a broad range of types of nodules.

Also in 2010, Gomathi and Thangaraj [54] used image processing techniques, Fuzzy C-Mean algorithm and neural classifier in the stages of preprocessing, segmentation and nodules detection, respectively. This system had an efficiency of 76.9% and 122 false positives being validated with 13 nodules and 8 nodules were less than 2 mm size.

Gavrielides et al. [73] presented a technique based on an adaptive filter to estimate the size of the nodules and investigated which were the interrelated factors that affect the accuracy in the measurement of pulmonary nodules. The main contribution of this paper is to present the main sources of error found in the measurement of pulmonary nodule, which may result in the appearance of new techniques. However, this research was restricted to solid nodules. Stefano Diciotti et al. [74] developed another approach aimed at measuring the size of the nodules, through the space scale Laplacian of Gaussian. The authors performed a validation of the method on in vitro and in vivo and results ensured the applicability of this approach.

In 2011, Kumar et al. [75] presented a CADe system that used Biorthogonal Wavelet Transform, region growing and fuzzy inference system in preprocessing, segmentation and detection of nodules, respectively. This system had a different approach, it not only determined the presence of nodules but also classified them into benign nodule (granuloma, hamartoma, for example), malignant neoplasia or malignant neoplasia in advanced stage. The system had a sensitivity of 86% and 2.17 FP per case being validated with 538 nodules. That same year, Tan et al. [76] developed a CADe system that used thresholding filter, rules and artificial neural network to segment images, detect nodules and elimination of false positive, respectively. They obtained a sensitivity of 87.5% with an average of 4 FP per case being tested with 574 nodules (isolated, juxtavascular, and juxtapleural) with diameters between 3mm and 30mm.

In 2012, Hong, Li and Yang [22] used Wiener and morphological filters with thresholding in the preprocessing and segmentation stages, respectively. For detection of candidate nodules, adaptive thresholding was used and SVM to eliminate false positives. This system had a sensitivity of 89.47% with 11.9 FP per case when tested with 44 solitary pulmonary nodules. The disadvantage of this approach is that the detection was restricted to solitary pulmonary nodules. Cascio et al. [65] made use of a neural classifier, region growing technique with morphological filter and Mass-spring models so that to eliminate false nodules, segment images of the lung and of the candidate nodules, respectively. The system achieved a performance of 97% with 6.1 FP per case being validated with 148 internal and juxtapleural nodules. In the same year, Orozco et al. [63] presented a system that used the Discrete Cosine Transform and the Fast Fourier Transform to determine the characteristics of texture and support vector machines for detecting pulmonary nodules. This system had a sensitivity of 96.15% with 2 FP per case when evaluated with 50 nodules. The disadvantage of this approach is that the image segmentation is performed manually. Moreover, the high value of sensitivity may not be representative, since this system was trained with only 50 nodules.

Ashwin et al. [19] developed a CADe system that used multilevel-thresholding growing and artificial neural networks in the stages of segmentation and detection of pulmonary nodules. This system achieved an accuracy of 96%. However, this system has only been tested with 40 cases, including the training and validation. Moreover, the authors did not report the size and location of the nodules tested. Chen et al. [77] carried out a study to compare the performances of the techniques of artificial neural networks (ANN) and multivariate logistic regression applied in differentiating between malignant and benign pulmonary nodules in CT images. As a result, artificial neural network achieved better performance with the accuracy rate of 90% when tested with 135 malignant nodules and 65 benign nodules.

In 2013, Teramoto and Fujita [24] proposed a detection method that prioritizes quick response. They used cylindrical filters and support vector machine to eliminate false positives with only seven parameters. The system obtained a sensitivity of 80% with 4.2 FP per case when validated with 103 nodules (juxtavascular, isolated, ground-glass opacity and juxtapleural), with diameters between 5mm and 20mm. The system showed a detection speed of 25-34 seconds per case, using a personal computer with 2.8 GHz processor.

The principal methods of detection of lung nodules are summarized in Table 2, through a comparison of the sensitivity, FP, number of nodules used in validation, size of nodules, response time and type of nodules.

**Table 2:** Performance comparison of lung nodule detection methods by sensitivity, FP, number of nodules, size and response time

**Methods**	**Year**	**Sensitivity**	**FP**	**N**^**°**^** of nodules**	**Size**	**Response time**	**Type of nodules**
Xu et al. [70]	1997	70%	1,7 per image	122	4 - 27mm	20s	NI
Armato et al. [48]	1999	70%	9,6 per case	187	3,1 - 27,8mm	NI	Solitary and juxtapleural
Lee et al. [45]	2001	72%	25,3 per case	98	< 10mm	187 min	NI
Suzuki et al. [71]	2003	80,3%	4,8 per case	121	4 - 27mm	1,4s	Juxtavascular, hilum, ground-glass opacity andjuxtapleural
Murphy et al. [40]	2007	84%	8,2 per case	268	2 - 14mm	NI	Pleural and non-pleural
Ye et al. [23]	2009	90,2%	8,2 per case	220	2 - 20mm	2,5 min	Juxtavascular, isolated, ground-glass opacityand juxtapleural
Messay, Hardie and Rogers [21]	2010	82,66%	3 per case	143	3 - 30mm	2,3 min	Juxtavascular, solitary, ground-glass opacityand juxtapleural
Liu et al. [20]	2010	97%	4,3 per case	32	NI	NI	Solitary
Kumar et al. [75]	2011	86%	2,17 per case	538	NI	NI	NI
Tan et al. [76]	2011	87,5%	4 per case	574	3 - 30mm	NI	Isolated, juxtavascular, and juxtapleural
Hong, Li and Yang [22]	2012	89,47%	11,9 per case	44	NI	NI	Solitary
Cascio et al. [65]	2012	97%	6,1 per case	148	≥ 3mm	1,5 min	Internal and juxtapleural
Orozco et al. [63]	2012	96,15%	2 per case	50	NI	NI	NI
Teramoto and Fujita [24]	2013	80%	4,2 per case	103	5 - 20mm	30s	Juxtavascular, isolated, ground-glass opacityand juxtapleural

(NI = Not Informed).

## Discussion

The use of CADe systems improves the performance of radiologists in the detection process of pulmonary nodules [10,11]. However, to be used routinely in the radiology department these systems must meet the following requirements: improve the performance of radiologists providing high sensitivity in the diagnosis, a low number of false positives, have high processing speed, present high level of automation, low cost (of implementation, training, support and maintenance), the ability to detect different types and shapes of nodules, and software security assurance.

Based on literature research, it was observed that many, if not all, systems described in this survey have the potential to be important in clinical practice. However, no significant improvement was observed in sensitivity, number of false positives, level of automation and ability to detect different types and shapes of nodules in the studied period. However, several systems showed promising results, for example, with regard to the parameters of sensitivity and number of FP, stood out the systems of Kumar et al. [75] and Tan et al. [76]. The first tested his method with 538 different nodules and had a sensitivity of 86% with 2.17 FP per case. The second validated his system with 574 different nodules and obtained a sensitivity of 87.5% with 4 FP per case. Other authors [20-22,24,45,63,65,71] also showed promising results, however, validation of these systems was limited to tests with up to 150 nodules. Thus, it is not guaranteed that these systems will present the same performance in other circumstances, because the system was not tested with a broad range of types of nodules.

On the issue of processing speed, although some authors have omitted this information, the systems generally showed satisfactory times that does not compromise their use in a clinical environment. Systems that showed faster response were Teramoto and Fujita [24], and Suzuki et al. [71] with the time of 30 seconds and 1.4 seconds, respectively. Several systems had a low level of automation because manual operations were needed. On the issue of ability to detect different types and shapes of nodules, stood out the systems of Suzuki et al. [71], Ye et al. [23], Messay, Hardie and Rogers [21], and Teramoto and Fujita [24] that detected juxtavascular, isolated, ground-glass opacity and juxtapleural nodules.

### Challenges

Further research is needed to improve existing systems and propose new solutions. For this, we believe that collaborative efforts through the creation of software communities are necessary to develop a CADe system with all the requirements mentioned and with a short development cycle. Thus, challenges for new CADe systems for detecting pulmonary nodules are: 

•Develop a CADe system, preferably an open source system, that will display all the functional requirements with zero cost of licensing and allowing to modify the source code according to local needs;

•Development of new techniques, or improve existing ones, of segmentation of lung images to allow higher level of automation, including cases of severe pathologies, small nodules (≤3*mm*) and with ground-glass opacity.

•Develop standards that allow to integrate CADe systems with other hospital environment systems (PACS and electronic patient record);

•Develop systems that identifies the nodules, determines their characteristics (malignancy, volume, presence of calcifications and their pattern, contours, edges and internal structures) and evaluates the evolution of the oncological therapy and its possible prognosis;

•Larger databases for efficient validation of proposed systems should be provided.

•The sensitivities of CADe systems are relatively high, but the number of FPs is high-compared to radiologists’ performance. Therefore, further improvement in specificity is necessary in future research.

## Conclusion

This paper presented a critical review of existing literature on Computer-Aided Detection systems for lung cancer in CT scans to identify challenges for future research. A systemic analysis was made on these articles and the results were summarized. No significant improvement was observed in sensitivity, number of false positives, level of automation and ability to detect different types and shapes of nodules in the studied period. However, several systems showed promising results.

These systems are not yet widely used in clinical practice, because most of these systems still require improvements to be accepted by the community of radiologists. Further research is needed to improve existing systems and propose new solutions. For this, we believe that collaborative efforts through the creation of free and open source software communities are necessary to develop a CADe system with all the requirements mentioned and with a short development cycle. In addition, future CADe systems should improve the level of automation, through integration with picture archiving and communication systems (PACS) and the electronic record of the patient, decrease the number of false positives, measure the evolution of tumors, evaluate the evolution of the oncological treatment, and its possible prognosis. I hope that this review will be useful for researchers to advance the development of CADe systems for lung cancer detection.

## Competing interests

The authors declare that they have no competing interests.

## Authors’ contributions

MF, AM and MRD: collection, organizing, and review of the literature; preparing the manuscript. RMM, HRH and RV: manuscript review, modification, editing, and revision. Both authors read and approved the final manuscript.
